# Hunger and Adherence to Antiretroviral Therapy: Learning From HIV Positive Caregivers of Orphans and Vulnerable Children in Tanzania

**DOI:** 10.3389/fpubh.2021.719485

**Published:** 2022-02-21

**Authors:** Amon Exavery, John Charles, Erica Kuhlik, Asheri Barankena, Ramadhani Abdul, Godfrey M. Mubyazi, Christina Kyaruzi, Levina Kikoyo, Elizabeth Jere, Marianna Balampama

**Affiliations:** ^1^PACT Tanzania, Dar es Salaam, Tanzania; ^2^Pact Inc., Washington, DC, United States; ^3^Impact Evaluation, Ifakara Health Institute, Dar es Salaam, Tanzania; ^4^Health Systems and Policy Research, National Institute for Medical Research, Dar es Salaam, Tanzania

**Keywords:** hunger, food security, HIV, antiretroviral therapy, adherence, caregivers

## Abstract

The association between hunger and adherence to antiretroviral therapy (ART) is less known especially in vulnerable populations receiving HIV care and treatment services. Caregivers of orphans and vulnerable children (OVC) are vulnerable and likely to experience hunger due to additional economic pressure in caring for OVC. Using data from the community–based, USAID–funded Kizazi Kipya project, this study assesses the association between hunger and ART adherence among caregivers of OVC in Tanzania. HIV positive caregivers enrolled in the project from January to July 2017 were analyzed. The outcome variable was adherence to ART, defined as “not having missed any ART dose in the last 30 days,” and household hunger, measured using the Household Hunger Scale (HHS), was the main independent variable. Data analysis included multivariable logistic regression. The study analyzed 11,713 HIV positive caregivers who were on ART at the time of enrollment in the USAID Kizazi Kipya project in 2017. Aged 48.2 years on average, 72.9% of the caregivers were female. While 34.6% were in households with little to no hunger, 59.4 and 6.0% were in moderate hunger and severe hunger households, respectively. Overall, 90.0% of the caregivers did not miss any ART dose in the last 30 days. ART adherence rates declined as household hunger increased (*p* < 0.001). Multivariable analysis showed that the odds of adhering to ART was significantly lower by 42% among caregivers in moderate hunger households than those in little to no hunger households (OR = 0.58, 95% CI 0.50–0.68). The decline increased to 47% among those in severe hunger households (OR = 0.53, 95% CI 0.41–0.69). Hunger is an independent and a significant barrier to ART adherence among caregivers LHIV in Tanzania. Improving access to adequate food as part of HIV care and treatment services is likely to improve ART adherence in this population.

## Introduction

Adherence to antiretroviral therapy (ART) is key to viral suppression for better health outcomes among people living with the Human Immunodeficiency Virus (PLHIV) (1–3). Research shows that adherence to ART improves immune recovery, decreases the incidence of non-AIDS events, comorbidities, and mortality, and reduces chances for HIV transmission (4–7). Adherence to ART also reduces rates of mother-to-child transmission (MTCT) of HIV (8–11). Suboptimal adherence to ART is detrimental: it is associated with virologic failure (12–19), drug resistance (12, 20–23), and ultimately death (24–30). In 2014, the United Nations Program on HIV/AIDS (UNAIDS) launched the 90-90-90 global targets for HIV and AIDS programming. The aim of the targets was that by 2020, 90% of all people living with HIV will know their status, 90% of people diagnosed with the HIV infection will be on ART, and 90% of people on ART will be virally suppressed (31). The targets were extended to 95-95-95 to be achieved by 2030 (32). Based on the principal of universal HIV testing and treatment of PLHIV, UNAIDS' theory is that HIV epidemic control is attainable when the 95-95-95 goals are reached. The central theme of the test and treat approach is that if people can be identified early on in their infection, start and stay on treatment (adherence) to achieve viral suppression, the onward transmission of HIV will be prevented, thus impacting HIV incidence at a population level (33). This is demonstrated by a large and growing body of clinical evidence showing that if an HIV positive person adheres to ART, the amount of HIV in the body (viral load) declines to a very low level, and when they are below 200 copies per milliliter of blood, a condition called “an undetectable viral load” (34), transmission of the HIV to others through sex or syringe sharing, and from mother to child during pregnancy, birth, and breastfeeding is prevented (34–37). In this context, undetectable HIV is said to be untransmittable (U = U).

Global estimates show that in 2017, there were 36.9 million PLHIV, 59.0% (21.7 million) of whom were accessing ART. Of those accessing ART, 81.0% had achieved viral suppression (i.e., fewer than 1,000 copies of HIV per milliliter of blood) (38). The same report also showed that in 2017, Africa accounted for 25.7 million of all PLHIV on the globe and only 61% were on treatment (38). In Tanzania, 1.4 million people aged 15 years and older were living with HIV in 2016/17. Of these, 60.6% knew their HIV status; 93.6% of those who knew their status self-reported being on ART; and 87.0% of those who reported being on ART were virally suppressed (39). This national viral load suppression achievement may have been contributed to by higher adherence rates which the country and development partners have continued to pursue. However, in view of the global emphasis of leaving no one behind in the HIV response (40), more efforts are still needed to achieve 100% ART adherence rate in all PLHIV. This is very crucial especially today when HIV treatment takes significant visibility in the HIV prevention strategies through the U = U (41). This study expands the evidence base by identifying barriers to ART adherence, which is one of the necessary components to achieving viral suppression (42, 43).

As ART adherence constitutes a precondition to viral suppression (44–46), client barriers to adherence should be assessed and addressed prior to starting a regimen (2). In this context, reports from many studies have identified predictors of poor adherence to ART such as financial constraints, forgetting to take the prescribed medications, lack of support from family members, as well as depression, alcohol consumption, social stigma and negative side effects of ART (47–55). Poor access to services, complex drug regimens, pregnancy, mental health disorders, substance abuse, weak social support networks and incarceration have also been identified (56). Other important factors associated with adherence to ART are reported to include sex (57), age, education, region of residence, episodic treatment, CD4 cell count, and the use of other prescribed drugs (58). In Tanzania, younger age, unemployment among adults aged 18 or more years (59), lack of bus fare for ART clinic attendance, alcohol use, and dissatisfaction with health providers (60) predicted non-adherence to ART.

While existing literature has suggested a possible link between food security and ART adherence, the relationship has not yet been rigorously examined or tested (61–64). A qualitative study in Uganda for example, observed five mechanisms through which food insecurity may contribute to ART non-adherence: (a) ART increases appetite, thus in the absence of food it leads to intolerable hunger; (b) in the absence of food, ART side effects heighten; (c) ART doses are skipped or not started at all if individuals believe that they cannot afford additional food costs; (d) competing demands between costs of food and medical expenses lead people either to default from treatment, or to give up food and wages to get medications; and (e) people sometimes forget to take their ART doses while working for food for long time in the fields (64).

Additionally, the evidence base for the relationship between food security and ART adherence is limited by small sample sizes and exclusion of vulnerable populations such as orphans and vulnerable children (OVC), and their caregivers (47, 50, 55, 57, 61, 65). Caregivers of OVC are also vulnerable and likely to experience food insecurity due additional economic pressure in caring for and meeting the needs of OVC (66). Therefore, studies that do not include OVC fail to examine the health, wellbeing, and skills of the caregivers, upon whom their health and wellbeing is highly dependent (67). Similarly, food security has not been sufficiently analyzed to clearly understand its relationship with ART adherence in this population.

To achieve control over the HIV epidemic, it is worthwhile to advance the evidence base for addressing barriers to service use in different populations. This study was undertaken to narrow the evidence gap by assessing the association between household hunger and adherence to ART among HIV positive caregivers of OVC receiving ART in Tanzania.

## Materials and Methods

### Data Source

This is a secondary analysis of data from Pact's community-based, USAID Kizazi Kipya project in Tanzania, with funding from the U.S. President's Emergency Plan for AIDS Relief (PEPFAR). The USAID Kizazi Kipya project (2016–2021) sought to increase the uptake of HIV services, other health, and social services by OVC and their caregivers. Data were collected during screening and enrollment of beneficiaries which was conducted by community case workers (CCWs) and lead case workers (LCWs) using the project's screening, enrollment, and Family and Child Asset Assessment (FCAA) tools between January–July 2017. Beneficiaries were enrolled in the project if their household met one or more enrollment criteria. The criteria are 14 HIV-related vulnerabilities published elsewhere (68).

Following beneficiary screening and enrollment, the USAID Kizazi Kipya project through its CCWs and LCWs developed a care plan for each OVC and the caregiver in the household. The care plan, among other things, constituted specific needs of each enrollee in the household. The project then provided or linked caregivers as well as OVC to health and social services including HIV, nutrition, education, child protection, social protection, and economic strengthening. The project directly provided psychosocial support, nutrition assessments, counseling, and support, referrals and linkages, and care plan monitoring (69). The project also tracked ART use and adherence among its HIV positive beneficiaries.

### Study Area

This study is based on enrollment data that were collected in 64 district councils in 19 regions of Tanzania where the USAID Kizazi Kipya project conducted beneficiary screening and enrollment activities during January–July 2017. The regions were: Dar es Salaam, Dodoma, Geita, Iringa, Kagera, Katavi, Mara, Mbeya, Mjini Magharibi, Morogoro, Mtwara, Mwanza, Njombe, Pwani, Rukwa, Ruvuma, Singida, Tabora, and Tanga.

### Study Design

This is a cross–sectional secondary analysis of existing enrollment data of the USAID Kizazi Kipya project described above. FCAA data were collected during screening and enrollment of beneficiaries. Beneficiaries from households meeting at least one of the enrollment criteria and consented to participate, were enrolled in the program. After enrollment, beneficiaries were followed up by the project team over time with provision of a variety of health, education, and other social services.

### Study Population

We analyzed data on 11,713 HIV-positive OVC caregivers who were living with HIV and on ART at the time of enrollment in the program. A caregiver is defined by the project as a guardian who has the greatest responsibility for the daily care and rearing of one or more OVC in a household. A caregiver is not necessarily a biological parent of the OVC in the household.

### Variables

Defined as not having missed any ART dose in the last 30 days preceding enrollment, adherence to ART was the only primary outcome investigated by the current study. It was self-reported by the respective caregiver, whereby, after establishing that the caregiver was LHIV and on ART, inquiries followed about whether the caregiver missed any dose in the previous 30 days. Ultimately, the variable was coded as “0” if the caregiver missed one or more doses of ART in the previous 30 days, and “1” if the caregiver did not miss any ART dose in the same period. This definition of adherence to ART as taking all pills prescribed, has been applied in another study (58).

Household hunger was the main independent variable for this study. The variable was constructed from the Household Hunger Scale (HHS), which was designed by the Food and Agriculture Organization (FAO) and the Tufts University through the Food and Nutrition Technical Assistance III Project (FANTA) (70). The HHS originates from the Household Food Insecurity Access Scale (HFIAS). It is a validated scale for cross-cultural use in food insecure settings. The HHS groups household hunger in three categories based on household hunger score: (a) little to no hunger, (b) moderate hunger, and (c) severe hunger (70).

Several other independent variables were collected, including caregiver's sex, level of formal education attained, marital status, family size, whether one or more family members has health insurance, whether the caregiver was physically or mentally disabled, place of residence (rural/urban), and household wealth quintile. Wealth quintile was constructed using principal component analysis (PCA) of household assets to obtain household socio–economic status (71). Five wealth quintiles were formed, ranging from the lowest quintile (Q1) for the poorest households, to the highest quintile (Q5) for the wealthiest households. Household assets included in the PCA process were: dwelling materials (brick, concrete, cement, aluminum, and/or other material), livestock (chicken, goats, cows, and others), transportation assets (bicycle, motorcycle/moped, tractor, motor vehicle, and others), and productive assets (sewing machine, television, couch or sofa, cooking gas, hair dryer, radio, refrigerator, blender, oven, and others).

### Data Analysis

An exploratory analysis was conducted through one-way tabulations of each variable to obtain distributional features of the data. Then, cross-tabulations of adherence to ART by each of the independent variables was conducted. For each pair of variables cross-tabulated, the purpose was to assess the variability in the rate of adherence to ART by levels of the independent variables. Since all variables were categorical, Chi-Square test was used to assess the significance of association between adherence to ART and each of the independent variables. Finally, binary logistic regression model was applied in the multivariable analysis to examine the association between household hunger on ART adherence, while adjusting for appropriate independent variables. Variables were considered eligible to be independent variables if their relevance with respect to ART adherence was justified in the literature, or if considered important at the program level. Then, apart from household hunger which was the main independent variable, other independent variables were included in the multivariable analysis if each one's presence improved the overall model. In all these analyses, cases of missing data were rare, hence excluded. Stata statistical software (version 14.0) was used for all the analyses, and all statistical inferences were made at the significance level of 5% (α = 0.05).

### Ethical Considerations

Ethics approval was received from the Medical Research Coordinating Committee (MRCC) of the National Institute for Medical Research (NIMR) in Tanzania (NIMR/HQ/R.8a/Vol.IX/3024). Enrollment of beneficiaries in the USAID Kizazi Kipya project was entirely voluntary. The FCAA tool was completed only after each participant signed a statement of informed consent. The USAID Kizazi Kipya project data are handled and stored very securely and confidentially.

## Results

### Respondents' Characteristics

The analysis was based on 11,713 HIV-positive caregivers aged at least 19 years (mean = 48.2) who were already on ART at the time of enrollment in the project. Of these, 72.9% were female and the rest were male. With respect to food security, while 34.6% were in households with little to no hunger, 59.4 and 6.0% were in moderate hunger and severe hunger households, respectively. In terms of marital status, 41.9% of the caregivers were widowed and 38.4% were married or living together. Primary education represented majority of the caregivers (78.8%). Majority of the caregivers (80.8%) did not have health insurance. Also, majority (75.8%) of the caregivers resided in rural areas. Further background details of the respondents are presented in Table 1.

**Table 1:** Profile of respondents (*n* = 11,713).

**Variable**	**Number of respondents (*n*)**	**Percent of respondents (%)**
**Level of household hunger**
Little to no hunger	4,055	34.6
Moderate hunger	6,953	59.4
Severe hunger	705	6.0
**Caregiver sex**		
Female	8,535	72.9
Male	3,178	27.1
**Age group**
18–29 years	337	2.9
30–39 years	2,241	19.1
40–49 years	4,385	37.4
50–59 years	2,890	24.7
60+ years	1,860	15.9
Mean = 48.2, SD = 11.5	—	—
**Marital status**
Married or living together	4,500	38.4
Divorced or separated	1,646	14.1
Never been married	658	5.6
Widowed	4,909	41.9
**Education attained**
Never been to school	2,183	18.6
Primary	9,232	78.8
Secondary or higher	298	2.6
**Family size**
2–3 people	4,416	37.7
4–6 people	5,386	46.0
7+ people	1,911	16.3
**Wealth quintile**
Lowest (Q1)	3,296	28.1
Second	937	8.0
Middle	1,928	16.5
Fourth	2,482	21.2
Highest (Q5)	3,070	26.2
**Place of residence**
Rural	8,882	75.8
Urban	2,831	24.2
**Mentally or physically disabled?**
No	11,353	96.9
Yes	360	3.1
**Family has health insurance (CHF/TIKA)**
No	9,465	80.8
Yes	2,248	19.2

### Adherence to ART by Level of Hunger and Other Characteristics

**Figure 1:**
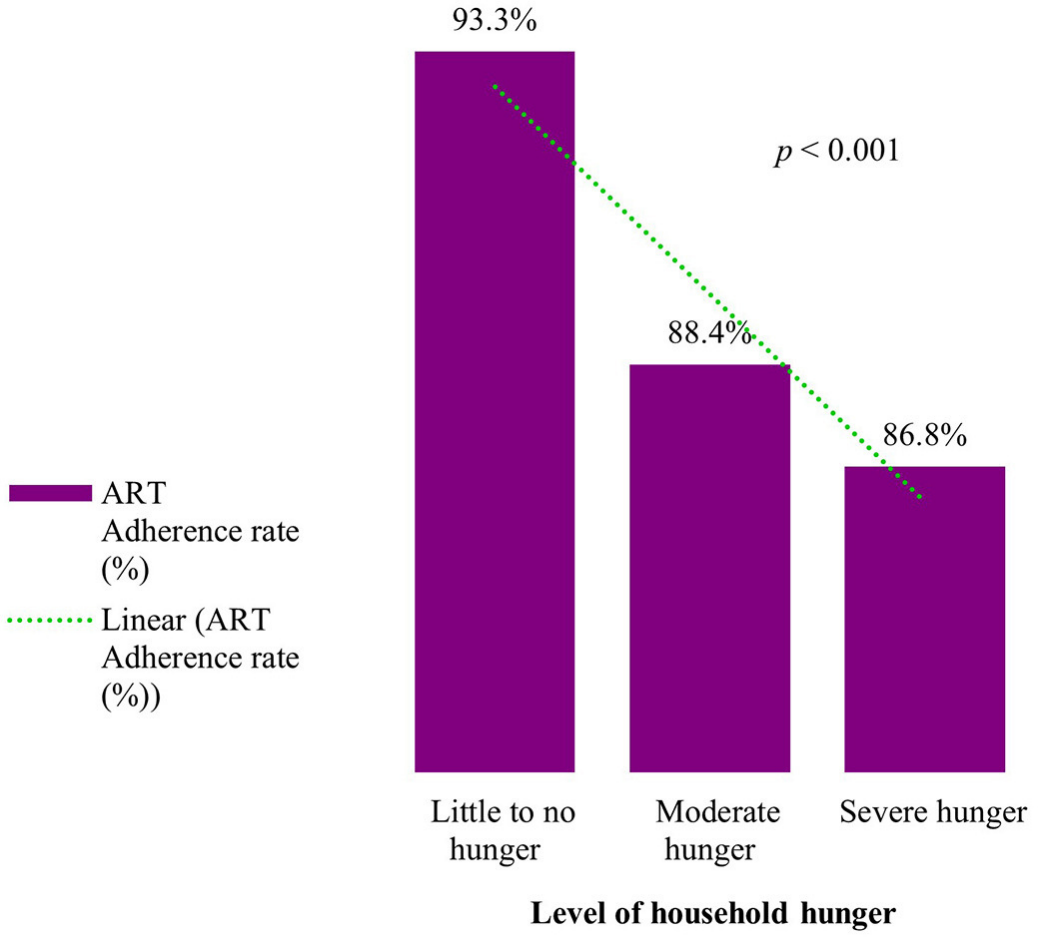
ART Adherence rate by level of household hunger among HIV positive OVC caregivers receiving ART in Tanzania (*n* = 11,713).

Overall, 90.0% of the caregivers were adherent to ART—that is, they had not missed any ART dose in the last 30 days preceding enrollment. The level of adherence to ART was constantly declining as household hunger increased: the highest adherence level was 93.3% among caregivers from households with little to no hunger, declined to 88.4% among caregivers in households with moderate hunger, and further declined to the lowest level of 86.8% among those who were in severe hunger households (*p* < 0.001) (Figure 1). Other factors which were significantly associated with ART adherence among OVC caregivers were: marital status (*p* < 0.001), age group (*p* < 0.001), wealth quintile (*p* = 0.001), place of residence (*p* < 0.001), education (*p* = 0.004), and ownership of health insurance (*p* = 0.002) (Table 2).

**Table 2:** Caregivers' adherence to ART by background characteristics (*n* = 11,713).

**Characteristic**	**% Caregivers adherent to ART**	*p-*value^*^
**Overall**	90.0	**—**
**Caregiver sex**		0.555
Female	89.9	
Male	90.3	
**Age group**		<0.001
18–29 years	82.8	
30–39 years	89.7	
40–49 years	90.5	
50–59 years	90.7	
60+ years	89.2	
**Marital status**		<0.001
Married or living together	90.6	
Divorced or separated	87.1	
Never been married	88.3	
Widowed	90.6	
**Education attained**		0.004
Never been to school	88.0	
Primary	90.4	
Secondary or higher	90.6	
**Family size**		0.409
2–3 people	90.2	
4–6 people	89.6	
7+ people	90.5	
**Wealth quintile**		0.001
Lowest (Q1)	88.1	
Second	90.4	
Middle	91.1	
Fourth	90.7	
Highest (Q5)	90.7	
**Place of residence**		<0.001
Rural	91.1	
Urban	86.5	
**Mentally or physically disabled?**		0.112
No	90.1	
Yes	87.5	
**Family has health insurance (CHF/TIKA)**		0.002
No	90.4	
Yes	88.2	

* *p-values are based on Pearson's Chi-Square test*.

### Results From Multivariable Analysis

Results from multivariable analysis (Table 3) showed that the odds of being adherent to ART was significantly and independently lower by 42% among the caregivers from moderate hunger households than those in little to no hunger households (OR = 0.58, 95% CI 0.50–0.68, *p* < 0.001). Caregivers from severe hunger households were 47% less likely to be adherent to ART than those from little to no hunger households (OR = 0.53, 95% CI 0.41–0.69, *p* < 0.001). These observations were adjusted for caregiver age, sex, marital status, education, family size, place of residence, health insurance, and wealth quintile.

**Table 3:** Multivariable logistic regression of the association between hunger and adherence to ART among HIV positive caregivers of orphans and vulnerable children receiving ART in Tanzania, 2017 (*n* = 11,713).

**Covariate**	**Adjusted odds ratio (OR)**	**95% Confidence interval**		***p*-value**
		**Lower limit**	**Upper limit**	
**Level of household hunger**
Little to no hunger	1.00	—	—	—
Moderate hunger	0.58	0.50	0.68	<0.001
Severe hunger	0.53	0.41	0.69	<0.001
**Caregiver sex**
Female	1.00	—	—	—
Male	0.89	0.77	1.04	0.827
**Age group**
18–29 years	1.00	—	—	—
30–39 years	1.76	1.28	2.42	0.002
40–49 years	1.92	1.41	2.61	<0.001
50–59 years	1.97	1.43	2.71	<0.001
60+ years	1.71	1.23	2.38	0.004
**Marital status**
Married or living together	1.00	—	—	—
Divorced or separated	0.74	0.61	0.89	0.009
Never married	0.96	0.73	1.26	0.713
Widowed	1.00	0.86	1.17	0.987
**Education attained**
Never been to school	1.00	—	—	—
Primary	1.30	1.12	1.52	0.011
Secondary or higher	1.53	1.01	2.33	0.040
**Family size**
2–3 people	1.00	—	—	—
4–6 people	0.91	0.80	1.05	0.899
7+ people	1.00	0.83	1.20	0.996
**Wealth quintile**				
Lowest (Q1)	1.00	—	—	—
Second	1.08	0.85	1.39	0.127
Middle	1.22	1.01	1.49	0.038
Fourth	1.13	0.95	1.36	0.115
Highest (Q5)	1.07	0.90	1.28	0.331
**Place of residence**
Rural	1.00	—	—	—
Urban	0.60	0.52	0.70	<0.001
**Mentally or physically disabled?**
No	1.00	—	—	—
Yes	0.84	0.61	1.16	0.077
**Family has health insurance (CHF/TIKA)**
No	1.00	—	—	—
Yes	0.67	0.57	0.77	<0.001

Also, Table 3 revealed other factors with significant association with adherence to ART among OVC caregivers in Tanzania. This included age, whereby the likelihood of adhering to ART was significantly higher among older caregivers (for each of the age categories) than those aged 18–29 years (30–39 years: OR = 1.76, 95% CI 1.28–2.42, *p* = 0.002; 40–49 years: OR = 1.92, 95% CI 1.41–2.61, *p* < 0.001; 50–59 years: OR = 1.97, 95% CI 1.43–2.71, *p* < 0.001; and 60+ years: OR = 1.71, 95% CI 1.23–2.38, *p* = 0.004). With respect to marital status, caregivers who were divorced or separated were significantly 26% less likely to be adherent to ART than those who were married or living together with their spouses (OR = 0.74, 95% CI 0.61–0.89, *p* = 0.009). Adherence among the rest of the marital categories were not significantly different from those who were married. The likelihood of adherence to ART among the caregivers increased with increasing levels of formal education attained compared to no education (primary education: OR = 1.30, 95% CI 1.12–1.52, *p* = 0.011; secondary or higher education: OR = 1.53, 95% CI 1.01–2.33, *p* = 0.040). Caregivers with health insurance were 33% less likely to be adherent to ART than those without (OR = 0.67, 95% CI 0.57–0.77, *p* < 0.001). Geographical variability in the adherence to ART was also observed, in which the likelihood of adherence to ART was 40% lower in urban than in rural areas (OR = 0.60, 95% CI 0.52–0.70, *p* < 0.001).

## Discussion

This study assessed the association between hunger and adherence to ART among HIV positive OVC caregivers receiving ART in Tanzania. As highlighted before, adherence to ART is a prerequisite for viral suppression and achieving better health outcomes among PLHIV (1, 3). In the current population, adherence to ART which was defined as not having missed any ART dose in the last 30 days, stood at 90.0%. This proportion was comparable with the 94.1% observed in another study in Tanzania that took place at an urban, faith-based clinic that was providing free ART to patients with a CD4 <200 (72). The recent Tanzania HIV Impact Survey found that 87.0% of PLHIV in Tanzania who are aged 15 years and older who self-report being on ART are virally suppressed (39). While much work has been done to achieve high rates of ART uptake and viral load suppression, a gap remains in attaining the UNAIDS 95-95-95 goals which must be met and maintained in order to achieve control of the HIV epidemic.

Multivariable analysis in the current study revealed declining likelihoods of ART adherence as the level of household hunger increased. Specifically, caregivers in moderate hunger households were 42% less likely than those in little to no hunger households to be adherent to ART (OR = 0.58, 95% CI 0.50–0.68). This increased to 47% among caregivers in severe hunger households than those in little to no hunger households (OR = 0.53, 95% CI 0.41–0.69). This observation is consistent with one systematic review (73), as well as other studies conducted among the general population in the Democratic Republic of Congo (74) and Cameroon (75). In all these studies, it was found that a lack of regular meals predicts non-adherence to ART (74, 75). Three studies in the systematic review observed that ART adherence was significantly better among recipients of food assistance than non-recipients (73). Therefore, to increase rates of ART adherence for better health outcomes among PLHIV, hunger or food insecurity should be appropriately addressed as an imperative element in HIV care and treatment programs.

Qualitative literature suggests that food security and adherence to ART may be related through several pathways, including the reasons that ART increases appetite and causes hunger (61–64, 76, 77), in the absence of food, antiretrovirals' side effects become intense (61, 64, 78), ART costs reduce household budgets available for food or nutritional needs (64), and forgetting to take antiretrovirals while working to meet food needs (64). Although the literature reveals these linkages, further research is needed to explore which of these pathways, or a combination of factors is most relevant to OVC caregivers and other vulnerable populations.

In addition, this study identified other factors (i.e., confounders) with a significant association with ART adherence among the caregivers. Age played a significant role, in which the likelihood of adherence to ART increased with age. This finding was consistent with another study in Ethiopia (79) and a collection of findings from one systematic review that found that older adults (age 50 years and above) living with HIV have a reduced risk of non-adherence to ART compared to younger adults (80). In the current study, adherence was lowest at 82.8% among younger adults under 30 years of age, suggesting the need to target this group with additional support to enhance ART adherence among them. The positive association between ART adherence and age has been shown in other studies, including longitudinal studies (81, 82). A possible explanation for this observation may be that people need time to comprehend the value of ART for them to maintain adherence. Alternatively, it may be that those individuals who do not adhere to treatment are more likely to be lost to follow-up from studies or die from AIDS-related illness, and thus by older ages only those who have maintained higher adherence rates remain in the sample. However, the current study cannot specifically ascertain a pathway through which the observed improvement in ART adherence with age functions. Therefore, further research is required to clarify socio–demographic, biologic, or any other means through which advancement in age improves adherence to ART among OVC caregivers LHIV in Tanzania.

With respect to marital status, the current study found that, caregivers who were currently divorced or separated were significantly 26% less likely than those who were married or living together with their spouses to be adherent to ART. ART adherence was highest among people in marital unions, suggesting that the extent of social support and encouragement available to the HIV patient may be better in marital unions. Social support may improve adherence as it provides psycho-social support and social accountability for the patient to remain on ART. While decrease in ART adherence due to lack of social support has been observed on one hand, living with a partner has been associated with increased social support and optimal adherence to ART on the other (83, 84). Similarly, improved adherence to ART in marriage has been inferred in other studies (75, 85, 86). This highlights the need to provide additional adherence support, including provision of emotional support to those outside marital unions or those without social support as a possible mechanism to improve adherence to ART among them.

Also, this study observed that higher levels of formal education attained were associated with higher likelihood of ART adherence among the caregivers. This was also concluded in other studies (53, 87). This finding was expected as those with better education are well-positioned to understand information related to health care, hence better adherence among them because they are able to follow ART instructions provided by health providers. Therefore, OVC caregivers who have never been to schools should be targeted with additional support to enhance ART adherence among them.

In terms of place of residence, the current study found higher adherence to ART in rural than in urban areas. This finding was consistent with that from another study in South Africa that indicated a higher rate of increased adherence to highly active ART among the populations living in rural areas than those in urban areas (81). Similarly, in the Unites States of America, another study found higher adherence in rural-small town or remote setting when compared to urban one (82). Although different reasons for this can be presented, it is possible that the communal nature of rural populations may have positive impacts of ART on PLHIV (81) through the increased assistance of one another, as well as recognizing and responding to each other's challenges and needs (88). This finding of higher ART adherence among rural populations suggests a need for ongoing adherence counseling support to sub–populations living in certain geographical settings such as those residing in urban areas.

Caregivers with health insurance were 33% less likely to be adherent to ART than those without the health insurance. Before the USAID Kizazi Kipya project, most of the communities in the study area were receiving support from another project called Pamoja Tuwalee, which among other things facilitated acquisition of health insurance, prioritizing destitute households. Given this, we conducted a separate analysis and found that more of the caregivers with health insurance were food insecure, and more than 50% of the caregivers who were food insecure were in the two lowest wealth quintiles. Therefore, caregivers with health insurance were mostly those from food insecure households, or socioeconomically the poorest. Although health insurance improves access to health services (89–91), its relationship with adherence to ART requires further research to reveal underlying mechanisms.

### Strengths and Limitations

Major strengths of this study exist in the use of a large sample size of OVC caregivers drawn from many regions of Tanzania, hence making it more of a nationally representative study. This makes the results applicable to the whole of Tanzania and possibly in other countries and settings with similar contexts. The use of standardized data collection tools reduced the possibility of information bias in this study.

However, this study has some limitations. Adherence to ART was assessed as a self-reported measure. This may have caused a recall bias in this study. However, during enrollment, efforts were made to minimize this through several measures, including short recall period (i.e., 1 month), good rapport between the caregivers (i.e., respondents) and the CCWs and LCWs, and thorough probes. Findings suggest that the bias may have been minimal because results are comparable with those from studies that measured adherence objectively. There is a possibility of residual confounding as some potential confounders observed in one systematic review, namely, alcohol consumption, use of traditional medicine, discrimination and stigmatization (92) were not available for inclusion in the multivariable analysis for this study. Had we had these variables, the magnitude of the association between household hunger and adherence to ART would probably have been different.

## Conclusions

Hunger is an independent and a significant barrier to ART adherence among OVC caregivers LHIV in Tanzania. This suggests that improving access to adequate food or provision of food supplementation as part of HIV care and treatment services is likely to improve adherence to ART among OVC caregivers and other vulnerable populations in resource limited settings.

In addition, to enhance ART adherence toward universal coverage, additional efforts may be required, to addresses contextual challenges related to geographical location of residences, younger ages especially below 30 years, and lack of formal education.

## Data Availability Statement

The raw data supporting the conclusions of this article will be made available by the authors, without undue reservation.

## Ethics Statement

The studies involving human participants were reviewed and approved by Medical Research Coordinating Committee (MRCC), National Institute for Medical Research (NIMR), Dar es Salaam, Tanzania. The patients/participants provided their written informed consent to participate in this study.

## Author Contributions

AE conceptualized the study, performed statistical analysis, drafted the initial version of the manuscript, and revised subsequent versions. JC refined the problem, participated in statistical analysis, and critically reviewed the manuscript. EK participated in writing and proofreading the manuscript. AB, RA, GMM, CK, LK, EJ, and MB critically reviewed the manuscript for intellectual content. All authors reviewed and approved the final manuscript.

## Funding

The USAID Kizazi Kipya is a 5-year project (from 2016 to 2021) implemented in Tanzania by Pact Tanzania as a prime organization, with funding from the U.S. President's Emergency Plan for AIDS Relief (PEPFAR) through the United States Agency for International Development (USAID) under the Cooperative Agreement No. (621-A-16-00001). The manuscript was produced as a part of authors' employment functions.

## Conflict of Interest

EK was employed by Pact, Inc. The remaining authors declare that the research was conducted in the absence of any commercial or financial relationships that could be construed as a potential conflict of interest.

## Publisher's Note

All claims expressed in this article are solely those of the authors and do not necessarily represent those of their affiliated organizations, or those of the publisher, the editors and the reviewers. Any product that may be evaluated in this article, or claim that may be made by its manufacturer, is not guaranteed or endorsed by the publisher.

## References

[1] Iacob SA Iacob DG Jugulete G. Improving the adherence to antiretroviral therapy, a difficult but essential task for a successful HIV treatment—clinical points of view and practical considerations. Front Pharmacol. (2017) 8:831. 10.3389/fphar.2017.00831 29218008 PMC5703840

[2] AIDSinfo. Adherence Limitations to Treatment Safety and Efficacy Adult and Adolescent ARV. (2017). *AIDSinfo*. Available online at: https://aidsinfo.nih.gov/guidelines/html/1/adult-and-adolescent-arv/30/adherence (accessed June 11, 2018).

[3] Hogg RS Heath KV Yip B Craib KJ O'Shaughnessy MV Schechter MT. Improved survival among HIV-infected individuals following initiation of antiretroviral therapy. JAMA. (1998) 279:450–4. 9466638 10.1001/jama.279.6.450

[4] Arkell C Harrigan M. HIV Treatment an Undetectable Viral Load to Prevent HIV Transmission. (2018). Available online at: http://www.catie.ca/en/fact-sheets/transmission/hiv-viral-load-hiv-treatment-and-sexual-hiv-transmission (accessed August 20, 2018).

[5] Granich RM Gilks CF Dye C De Cock KM Williams BG. Universal voluntary HIV testing with immediate antiretroviral therapy as a strategy for elimination of HIV transmission: a mathematical model. Lancet. (2009) 373:48–57. 10.1016/S0140-6736(08)61697-9 19038438

[6] Albert J Berglund T Gisslén M Gröön P Sönnerborg A Tegnell A. Risk of HIV transmission from patients on antiretroviral therapy: a position statement from the Public Health Agency of Sweden and the Swedish Reference Group for Antiviral Therapy. Scand J Infect Dis. (2014) 46:673–7. 10.3109/00365548.2014.926565 25073537 PMC4196576

[7] Fang C-T Hsu H-M Twu S-J Chen M-Y Chang Y-Y Hwang J-S. Decreased HIV transmission after a policy of providing free access to highly active antiretroviral therapy in Taiwan. J Infect Dis. (2004) 190:879–85. 10.1086/422601 15295691

[8] Agabu A Baughman AL Fischer-Walker C Klerk M de Mutenda N Rusberg F. National-level effectiveness of ART to prevent early mother to child transmission of HIV in Namibia. PLoS ONE. (2020) 15:e0233341. 10.1371/journal.pone.0233341 33170840 PMC7654758

[9] Mugwaneza P Lyambabaje A Umubyeyi A Humuza J Tsague L Mwanyumba F. Impact of maternal ART on mother-to-child transmission (MTCT) of HIV at six weeks postpartum in Rwanda. BMC Public Health. (2018) 18:1248. 10.1186/s12889-018-6154-6 30419870 PMC6233517

[10] Cooper ER Charurat M Mofenson L Hanson IC Pitt J Diaz C. Combination antiretroviral strategies for the treatment of pregnant HIV-1-infected women and prevention of perinatal HIV-1 transmission. J Acquir Immune Defic Syndr. (2002) 29:484–94. 10.1097/00126334-200204150-00009 11981365

[11] Guay LA Musoke P Fleming T Bagenda D Allen M Nakabiito C. Intrapartum and neonatal single-dose nevirapine compared with zidovudine for prevention of mother-to-child transmission of HIV-1 in Kampala, Uganda: HIVNET 012 randomised trial. Lancet. (1999) 354:795–802. 10.1016/S0140-6736(99)80008-7 10485720

[12] WHO. HIV Drug Resistance. (2021). Available online at: https://www.who.int/news-room/fact-sheets/detail/hiv-drug-resistance (accessed December 13, 2021).

[13] Zenu S Tesema T Reshad M Abebe E. Determinants of first-line antiretroviral treatment failure among adult patients on treatment in Mettu Karl Specialized Hospital, South West Ethiopia; a case control study. PLoS ONE. (2021) 16:e0258930. 10.1371/journal.pone.0258930 34679085 PMC8535443

[14] Lailulo Y Kitenge M Jaffer S Aluko O Nyasulu PS. Factors associated with antiretroviral treatment failure among people living with HIV on antiretroviral therapy in resource-poor settings: a systematic review and metaanalysis. Syst Rev. (2020) 9:292. 10.1186/s13643-020-01524-1 33308294 PMC7733304

[15] Chawana TD Reid A Bwakura T Gavi S Nhachi CFB. Factors influencing treatment failure in HIV positive adult patients on first line antiretroviral therapy. Cent Afr J Med. (2014) 60:29–36. 26867253

[16] Martin M Del Cacho E Codina C Tuset M De Lazzari E Mallolas J. Relationship between adherence level, type of the antiretroviral regimen, and plasma HIV type 1 RNA viral load: a prospective cohort study. AIDS Res Hum Retroviruses. (2008) 24:1263–8. 10.1089/aid.2008.0141 18834323

[17] Nachega JB Hislop M Dowdy DW Chaisson RE Regensberg L Maartens G. Adherence to nonnucleoside reverse transcriptase inhibitor-based HIV therapy and virologic outcomes. Ann Intern Med. (2007) 146:564–73. 10.7326/0003-4819-146-8-200704170-00007 17438315

[18] Maggiolo F Ravasio L Ripamonti D Gregis G Quinzan G Arici C. Similar adherence rates favor different virologic outcomes for patients treated with nonnucleoside analogues or protease inhibitors. Clin Infect Dis. (2005) 40:158–63. 10.1086/426595 15614706

[19] Paterson DL Swindells S Mohr J Brester M Vergis EN Squier C. Adherence to protease inhibitor therapy and outcomes in patients with HIV infection. Ann Intern Med. (2000) 133:21–30. 10.7326/0003-4819-133-1-200007040-00004 10877736

[20] Ekong E Ndembi N Okonkwo P Dakum P Idoko J Banigbe B. Epidemiologic and viral predictors of antiretroviral drug resistance among persons living with HIV in a large treatment program in Nigeria. AIDS Res Ther. (2020) 17:7. 10.1186/s12981-020-0261-z 32066473 PMC7027291

[21] Bangsberg DR Acosta EP Gupta R Guzman D Riley ED Harrigan PR. Adherence-resistance relationships for protease and non-nucleoside reverse transcriptase inhibitors explained by virological fitness. AIDS. (2006) 20:223–31. 10.1097/01.aids.0000199825.34241.49 16511415

[22] Harrigan PR Hogg RS Dong WWY Yip B Wynhoven B Woodward J. Predictors of HIV drug-resistance mutations in a large antiretroviral-naive cohort initiating triple antiretroviral therapy. J Infect Dis. (2005) 191:339–47. 10.1086/427192 15633092

[23] Sethi AK Celentano DD Gange SJ Moore RD Gallant JE. Association between adherence to antiretroviral therapy and human immunodeficiency virus drug resistance. Clin Infect Dis. (2003) 37:1112–8. 10.1086/378301 14523777

[24] Teshale AB Tsegaye AT Wolde HF. Incidence of mortality and its predictors among HIV positive adults on antiretroviral therapy in university of gondar comprehensive specialized hospital, Northwest Ethiopia. HIV AIDS (Auckl). (2021) 13:31–9. 10.2147/HIV.S289794 33469384 PMC7812522

[25] Haguihara T Silva MDO Rebouças MC Martins Netto E Brites C. Factors associated with mortality in HIV patients failing antiretroviral therapy, in Salvador, Brazil. Brazilian J Infect Dis. (2019) 23:160–3. 10.1016/j.bjid.2019.06.001 31301280 PMC9428215

[26] Akbari M Fararouei M Haghdoost AA Gouya MM Kazerooni PA. Survival and associated factors among people living with HIV/AIDS: a 30-year national survey in Iran. J Res Med Sci. (2019) 24:5. 10.4103/jrms.JRMS_630_18 30815018 PMC6383342

[27] Biset Ayalew M. Mortality and its predictors among HIV infected patients taking antiretroviral treatment in Ethiopia: a systematic review. AIDS Res Treat. (2017) 2017:5415298. 10.1155/2017/5415298 29214077 PMC5682904

[28] Lima VD Harrigan R Bangsberg DR Hogg RS Gross R Yip B. The combined effect of modern highly active antiretroviral therapy regimens and adherence on mortality over time. J Acquir Immune Defic Syndr. (2009) 50:529–36. 10.1097/QAI.0b013e31819675e9 19223785 PMC3606956

[29] Nachega JB Hislop M Dowdy DW Lo M Omer SB Regensberg L. Adherence to highly active antiretroviral therapy assessed by pharmacy claims predicts survival in HIV-infected South African adults. J Acquir Immune Defic Syndr. (2006) 43:78–84. 10.1097/01.qai.0000225015.43266.46 16878045

[30] García de Olalla P Knobel H Carmona A Guelar A López-Colomés JL Caylà JA. Impact of adherence and highly active antiretroviral therapy on survival in HIV-infected patients. J Acquir Immune Defic Syndr. (2002) 30:105–10. 10.1097/00042560-200205010-00014 12048370

[31] UNAIDS. 90-90-90: An Ambitious Treatment Target to Help End the AIDS Epidemic. Geneva: UNAIDS (2014).

[32] UNAIDS. Fast Track: Ending the AIDS Epidemic by 2030. Geneva: UNAIDS (2014).

[33] Gray G. HIV, AIDS 90-90-90: What is it Why Does it Matter? The Conversation. (2016). Available online at http://theconversation.com/hiv-aids-and-90-90-90-what-is-it-and-why-does-it-matter-62136 (accessed August 15, 2019).

[34] Cohen MS Chen YQ McCauley M Gamble T Hosseinipour MC Kumarasamy N. Antiretroviral therapy for the prevention of HIV-1 transmission. N Engl J Med. (2016) 375:830–9. 10.1056/NEJMoa1600693 27424812 PMC5049503

[35] Rodger AJ Cambiano V Bruun T Vernazza P Collins S van Lunzen J. Sexual activity without condoms and risk of HIV transmission in serodifferent couples when the HIV-positive partner is using suppressive antiretroviral therapy. JAMA. (2016) 316:171–81. 10.1001/jama.2016.5148 27404185

[36] Bavinton BR Pinto AN Phanuphak N Grinsztejn B Prestage GP Zablotska-Manos IB. Viral suppression and HIV transmission in serodiscordant male couples: an international, prospective, observational, cohort study. Lancet HIV. (2018) 5:e438–47. 10.1016/S2352-3018(18)30132-2 30025681

[37] CDC. Evidence of HIV Treatment and Viral Suppression in Preventing the Sexual Transmission of HIV. (2018). Available online at: https://www.cdc.gov/hiv/pdf/risk/art/cdc-hiv-art-viral-suppression.pdf (accessed June 14, 2019).

[38] UNAIDS. Global HIV and AIDS Statistics — 2018 Fact Sheet. (2018). Available online at: https://www.unaids.org/en/resources/fact-sheet (accessed July 17, 2019).

[39] Tanzania Commission for AIDS (TACAIDS) Zanzibar AIDS Commission (ZAC). Tanzania HIV Impact Survey (THIS) 2016-2017: Final Report. Dar es Salaam: Tanzania Commission for AIDS (TACAIDS); Zanzibar AIDS Commission (ZAC) (2018).

[40] WHO. Progress Report 2016 Prevent HIV, Test and Treat All: WHO Support for Country Impact. Geneva: WHO (2016).

[41] Okoli C Van de Velde N Richman B Allan B Castellanos E Young B. Undetectable equals untransmittable (U = U): awareness and associations with health outcomes among people living with HIV in 25 countries. Sex Transm Infect. (2021) 97:18–26. 10.1136/sextrans-2020-054551 32732335 PMC7841488

[42] Shrestha R Copenhaver MM. Viral suppression among HIV-infected methadone-maintained patients: the role of ongoing injection drug use and adherence to antiretroviral therapy (ART). Addict Behav. (2018) 85:88–93. 10.1016/j.addbeh.2018.05.031 29879611 PMC6337064

[43] Byrd KK Hou JG Bush T Hazen R Kirkham H Delpino A. Adherence and viral suppression among participants of the Patient-centered HIV Care Model project-a collaboration between community-based pharmacists and HIV clinical providers. Clin Infect Dis. (2020) 70:789–97. 10.1093/cid/ciz276 30953062 PMC6821576

[44] Maina EK Mureithi H Adan AA Muriuki J Lwembe RM Bukusi EA. Incidences and factors associated with viral suppression or rebound among HIV patients on combination antiretroviral therapy from three counties in Kenya. Int J Infect Dis. (2020) 97:151–8. 10.1016/j.ijid.2020.05.097 32497804

[45] Lokpo SY Ofori-Attah PJ Ameke LS Obirikorang C Orish VN Kpene GE. Viral suppression and its associated factors in HIV patients on highly active antiretroviral therapy (HAART): a retrospective study in the Ho Municipality, Ghana. AIDS Res Treat. (2020) 2020:e9247451. 10.1155/2020/9247451

[46] Arnsten JH Demas PA Farzadegan H Grant RW Gourevitch MN Chang C-J. Antiretroviral therapy adherence and viral suppression in HIV-infected drug users: comparison of self-report and electronic monitoring. Clin Infect Dis. (2001) 33:1417–23. 10.1086/323201 11550118 PMC2692641

[47] Achappa B Madi D Bhaskaran U Ramapuram JT Rao S Mahalingam S. Adherence to antiretroviral therapy among people living with HIV. N Am J Med Sci. (2013) 5:220–3. 10.4103/1947-2714.109196 23626959 PMC3632027

[48] Li L Lee S-J Wen Y Lin C Wan D Jiraphongsa C. Antiretroviral therapy adherence among patients living with HIV/AIDS in Thailand. Nurs Health Sci. (2010) 12:212–20. 10.1111/j.1442-2018.2010.00521.x 20602694 PMC2947817

[49] Meresse M March L Kouanfack C Bonono R-C Boyer S Laborde-Balen G. Patterns of adherence to antiretroviral therapy and HIV drug resistance over time in the Stratall ANRS 12110/ESTHER trial in Cameroon. HIV Med. (2014) 15:478–87. 10.1111/hiv.12140 24589279

[50] Obirikorang C Selleh PK Abledu JK Fofie CO. Predictors of adherence to antiretroviral therapy among HIV/AIDS patients in the Upper West Region of Ghana. Int Scholarly Res Notices. (2013) 2013:873939. 10.1155/2013/873939 24386593 PMC3872409

[51] Oku AO Owoaje ET Ige OK Oyo-Ita A. Prevalence and determinants of adherence to HAART amongst PLHIV in a tertiary health facility in south-south Nigeria. BMC Infect Dis. (2013) 13:401. 10.1186/1471-2334-13-401 24229404 PMC3765999

[52] Olowookere SA Fatiregun AA Akinyemi JO Bamgboye AE Osagbemi GK. Prevalence and determinants of nonadherence to highly active antiretroviral therapy among people living with HIV/AIDS in Ibadan, Nigeria. J Infect Dev Ctries. (2008) 2:369–72. 10.3855/jidc.199 19745505

[53] Pennap GR Abdullahi U Bako IA. Adherence to highly active antiretroviral therapy and its challenges in people living with human immunodeficiency virus (HIV) infection in Keffi, Nigeria. J AIDS HIV Res. (2013) 5:52–28. 10.5897/JAHR12.064

[54] Wakibi SN Ng'ang'a ZW Mbugua GG. Factors associated with non-adherence to highly active antiretroviral therapy in Nairobi, Kenya. AIDS Res Ther. (2011) 8:43. 10.1186/1742-6405-8-43 22141425 PMC3247823

[55] Yaya I Landoh DE Saka B Patchali PM Wasswa P Aboubakari A. Predictors of adherence to antiretroviral therapy among people living with HIV and AIDS at the regional hospital of Sokodé, Togo. BMC Public Health. (2014) 14:1308. 10.1186/1471-2458-14-1308 25526773 PMC4300825

[56] WHO. Consolidated Guidelines on the Use of Antiretroviral Drugs for Treating and Preventing HIV Infection: Recommendations for a Public Health Approach. Geneva: World Health Organization (2013). 24716260

[57] Polisset J Ametonou F Arrive E Aho A Perez F. Correlates of adherence to antiretroviral therapy in HIV-infected children in Lomé, Togo, West Africa. AIDS Behav. (2009) 13:23–32. 10.1007/s10461-008-9437-6 18654845

[58] O'Connor JL Gardner EM Mannheimer SB Lifson AR Esser S Telzak EE. Factors associated with adherence amongst 5295 people receiving antiretroviral therapy as part of an international trial. J Infect Dis. (2013) 208:40–9. 10.1093/infdis/jis731 23204161 PMC3666133

[59] Semvua SK Orrell C Mmbaga BT Semvua HH Bartlett JA Boulle AA. Predictors of non-adherence to antiretroviral therapy among HIV infected patients in northern Tanzania. PLoS ONE. (2017) 12:e0189460. 10.1371/journal.pone.0189460 29252984 PMC5734684

[60] Idindili B Jullu B Mugusi F Tanner M. A case-control study of factors associated with non-adherent to antiretroviral therapy among HIV infected people in Pwani Region, eastern Tanzania. Tanzan J Health Res. (2012) 14:194–203. 10.4314/thrb.v14i3.6 26591757

[61] Nagata JM Magerenge RO Young SL Oguta JO Weiser SD Cohen CR. Social determinants, lived experiences, and consequences of household food insecurity among persons living with HIV/AIDS on the shore of Lake Victoria, Kenya. AIDS Care. (2012) 24:728–36. 10.1080/09540121.2011.630358 22150119 PMC4418445

[62] Au JT Kayitenkore K Shutes E Karita E Peters PJ Tichacek A. Access to adequate nutrition is a major potential obstacle to antiretroviral adherence among HIV-infected individuals in Rwanda. AIDS. (2006) 20:2116–8. 10.1097/01.aids.0000247580.16073.1b 17053359

[63] Coetzee B Kagee A Vermeulen N. Structural barriers to adherence to antiretroviral therapy in a resource-constrained setting: the perspectives of health care providers. AIDS Care. (2011) 23:146–51. 10.1080/09540121.2010.498874 21259126

[64] Weiser SD Tuller DM Frongillo EA Senkungu J Mukiibi N Bangsberg DR. Food insecurity as a barrier to sustained antiretroviral therapy adherence in Uganda. PLoS ONE. (2010) 5:e10340. 10.1371/journal.pone.0010340 20442769 PMC2860981

[65] Wang X Wu Z. Factors associated with adherence to antiretroviral therapy among HIV/AIDS patients in rural China. AIDS. (2007) 21(Suppl. 8):S149–55. 10.1097/01.aids.0000304711.87164.99 18172384

[66] Karimli L Ssewamala FM Ismayilova L. Extended families and perceived caregiver support to AIDS orphans in Rakai district of Uganda. Child Youth Serv Rev. (2012) 34:1351–8. 10.1016/j.childyouth.2012.03.015 23188930 PMC3505487

[67] CARE. Promising Practices: Promoting Early Childhood Development for OVC in Resource Constrained Settings: The 5x5 Model. Geneva: Care, USAID, Hope for African Children Initiative (2006).

[68] Exavery A Charles J Kuhlik E Barankena A Koler A Kikoyo L. Understanding the association between caregiver sex and HIV infection among orphans and vulnerable children in Tanzania: learning from the USAID Kizazi Kipya project. BMC Health Serv Res. (2020) 20:1–14. 10.1186/s12913-020-05102-y 32245468 PMC7119283

[69] Pact. Kizazi Kipya: New Generation. Pact (2019). Available online at: http://www.pactworld.org/country/tanzania/project (accessed August 20, 2018).

[70] Ballard T Coates J Swindale A Deitchler M. Household Hunger Scale: Indicator Definition and Measurement Guide. Washington, DC: Food and Nutrition Technical Assistance II Project, FHI 360 (2011).

[71] Vyas S Kumaranayake L. Constructing socio-economic status indices: how to use principal components analysis. Health Policy Plan. (2006) 21:459–68. 10.1093/heapol/czl029 17030551

[72] Watt MH Maman S Golin CE Earp JA Eng E Bangdiwala S. Factors associated with self-reported adherence to antiretroviral therapy in a Tanzanian setting. AIDS Care. (2010) 22:381–9. 10.1080/09540120903193708 20390519 PMC3534771

[73] Singer AW Weiser SD McCoy SI. Does food insecurity undermine adherence to antiretroviral therapy? A systematic review. AIDS Behav. (2015) 19:1510–26. 10.1007/s10461-014-0873-1 25096896

[74] Musumari PM Wouters E Kayembe PK Kiumbu Nzita M Mbikayi SM Suguimoto SP. Food insecurity is associated with increased risk of non-adherence to antiretroviral therapy among HIV-infected adults in the Democratic Republic of Congo: a cross-sectional study. PLoS ONE. (2014) 9:e85327. 10.1371/journal.pone.0085327 24454841 PMC3893174

[75] Boyer S Clerc I Bonono C-R Marcellin F Bilé P-C Ventelou B. Non-adherence to antiretroviral treatment and unplanned treatment interruption among people living with HIV/AIDS in Cameroon: individual and healthcare supply-related factors. Soc Sci Med. (2011) 72:1383–92. 10.1016/j.socscimed.2011.02.030 21470734

[76] Irunde H Temu F Maridadi J Nsimba S Comoro C. A Study on Antiretroviral Adherence in Tanzania: a Pre-Intervention Perspective, 2005. (2006). Geneva: WHO.

[77] Murray LK Semrau K McCurley E Thea DM Scott N Mwiya M. Barriers to acceptance and adherence of antiretroviral therapy in urban Zambian women: a qualitative study. AIDS Care. (2009) 21:78–86. 10.1080/09540120802032643 19085223 PMC2821879

[78] Young S Wheeler AC McCoy SI Weiser SD. A review of the role of food insecurity in adherence to care and treatment among adult and pediatric populations living with HIV and AIDS. AIDS Behav. (2014) 18:505–15. 10.1007/s10461-013-0547-4 23842717 PMC3888651

[79] Letta S Demissie A Oljira L Dessie Y. Factors associated with adherence to antiretroviral therapy (ART) among adult people living with HIV and attending their clinical care, Eastern Ethiopia. BMC Int Health Hum Rights. (2015) 15:33. 10.1186/s12914-015-0071-x 26711659 PMC4693416

[80] Ghidei L Simone M Salow M Zimmerman K Paquin AM Skarf LM. Aging, antiretrovirals, and adherence: a meta analysis of adherence among older HIV-infected individuals. Drugs Aging. (2013) 30:809–19. 10.1007/s40266-013-0107-7 23959913 PMC3844933

[81] Maqutu D Zewotir T North D Naidoo K Grobler A. Determinants of optimal adherence over time to antiretroviral therapy amongst HIV positive adults in South Africa: a longitudinal study. AIDS Behav. (2011) 15:1465–74. 10.1007/s10461-010-9688-x 20352319 PMC3056165

[82] Ohl ME Perencevich E McInnes DK Kim N Rimland D Akgun K. Antiretroviral adherence among rural compared to urban veterans with HIV infection in the United States. AIDS Behav. (2013) 17:174–80. 10.1007/s10461-012-0325-8 23080359

[83] Williams A Friedland G. Adherence, compliance, and HAART. AIDS Clin Care. (1997) 9:51–4. 11364415

[84] Godin G Côté J Naccache H Lambert LD Trottier S. Prediction of adherence to antiretroviral therapy: a one-year longitudinal study. AIDS Care. (2005) 17:493–504. 10.1080/09540120412331291715 16036235

[85] Beyene KA Gedif T Gebre-Mariam T Engidawork E. Highly active antiretroviral therapy adherence and its determinants in selected hospitals from south and central Ethiopia. Pharmacoepidemiol Drug Saf. (2009) 18:1007–15. 10.1002/pds.1814 19650153

[86] Okoronkwo I Okeke U Chinweuba A Iheanacho P. Nonadherence factors and sociodemographic characteristics of HIV-infected adults receiving antiretroviral therapy in Nnamdi Azikiwe University Teaching Hospital, Nnewi, Nigeria. Int Scholarly Res Notices. (2013) 2013:843794. 10.1155/2013/843794 24369526 PMC3863502

[87] Wasti SP Simkhada P Randall J Freeman JV van Teijlingen E. Factors influencing adherence to antiretroviral treatment in Nepal: a mixed-methods study. PLoS ONE. (2012) 7:e35547. 10.1371/journal.pone.0035547 22563464 PMC3341373

[88] Pew Research Center. What Unites and Divides Urban, Suburban and Rural Communities. Washington, DC: Pew Research Center (2018).

[89] Hoffman C Paradise J. Health insurance and access to health care in the United States. Ann NY Acad Sci. (2008) 1136:149–60. 10.1196/annals.1425.007 17954671

[90] Richardson E Roberts B Sava V Menon R McKee M. Health insurance coverage and health care access in Moldova. Health Policy Plan. (2012) 27:204–12. 10.1093/heapol/czr024 21441565

[91] Amu H Dickson KS Kumi-Kyereme A Darteh EKM. Understanding variations in health insurance coverage in Ghana, Kenya, Nigeria, and Tanzania: evidence from demographic and health surveys. PLoS ONE. (2018) 13:e0201833. 10.1371/journal.pone.0201833 30080875 PMC6078306

[92] Heestermans T Browne JL Aitken SC Vervoort SC Klipstein-Grobusch K. Determinants of adherence to antiretroviral therapy among HIV-positive adults in sub-Saharan Africa: a systematic review. BMJ Global Health. (2016) 1:e000125. 10.1136/bmjgh-2016-000125 28588979 PMC5321378

